# Quality of life of primary caregivers by severity and control of children’s asthma: a systematic review and meta-analysis

**DOI:** 10.1007/s11136-025-04042-6

**Published:** 2025-08-27

**Authors:** Florian Tomini, Mathura Nagarajah, Sharumilan Ravindran, Borislava Mihaylova

**Affiliations:** 1 Health Economics and Policy Research Unit, Centre for Evaluation and Methods, Wolfson Institute of Population Health, Queen Mary University of London, London, E1 4NS UK; 2 Barts and The London School of Medicine and Dentistry, Queen Mary University of London, London, UK; 3 Health Economics Research Centre, Oxford Population Health, University of Oxford, Oxford, UK

**Keywords:** Asthma, Asthma severity, Asthma control, Paediatric, Caregivers, Quality of life, Systematic review

## Abstract

This systematic review and meta-analysis examined how the quality of life (QOL) of primary caregivers of children with asthma varies according to asthma severity and control. We also compared caregiver QOL with that of caregivers of healthy children and explored variation across specific QOL domains. A comprehensive search was conducted in PubMed, Embase, Scopus, and Web of Science from inception to October 2023. Eligible studies reported caregiver QOL in relation to asthma severity, asthma control, or healthy controls. Study quality was assessed using the Newcastle–Ottawa Scale. QOL scores were standardised to a 0–100 scale (0 = lowest, 100 = highest) and synthesised using random-effects meta-analysis. Thirteen studies were included. Caregivers of healthy children had the highest QOL (79.63; 95% CI: 69.51–89.75), while those of children with severe persistent asthma reported lower scores (59.63; 95% CI: 55.26–64.00). QOL was also higher among caregivers of children with good asthma control (76.71; 95% CI: 67.58–85.84) compared to those with poor control (62.48; 95% CI: 50.15–74.81). Emotional well-being and activity limitations were the domains most consistently associated with severity and control levels. Caregiver QOL is associated with asthma severity and control. Our findings underscore the need for consistent symptom monitoring and management, and suggest that caregiver support should be a routine consideration in paediatric asthma care.

**Supplementary Information:**

The online version contains supplementary material available at 10.1007/s11136-025-04042-6.

## Plain English summary

Taking care of a child with asthma can be really hard on parents and caregivers, especially if the child's asthma is continuously persistent or hard to control. Our study looked into how the severity of a child’s asthma affects the quality of life for those caring for them. We reviewed and combined the results of 13 existing studies that measured caregivers’ quality of life using standard tools.

The findings showed that caregivers of healthy children generally reported a better quality of life compared to those caring for children with severe or poorly controlled asthma. The biggest impacts were on their emotional well-being and limitations with everyday activities.

This review highlights the importance of managing a child’s asthma effectively, not only for the child's health but also for the family’s overall well-being. It emphasises the need to provide better support, education, and resources for caregivers, especially those caring for children with more severe asthma.

## Introduction

Asthma is a chronic medical condition that imposes a significant burden on individuals and healthcare systems, often leading to premature death or a substantial decline in quality of life [1]. Children and adolescents are particularly affected by asthma, experiencing higher disease frequency and severity compared to adults [2]. Severe asthma is typically defined by the necessity for high-intensity treatment or remaining uncontrolled despite such treatment [1, 3, 4].

In children, severe or uncontrolled asthma can significantly impair their quality of life (QOL) and necessitate ongoing management to alleviate symptoms and reduce the frequency of exacerbations [1]. Acute exacerbations are a common cause of hospital admissions in paediatric patients and can be life-threatening [5]. The impact of severe or uncontrolled asthma extends beyond the affected child, influencing the entire family, particularly parents and those primarily responsible for the day-to-day management of the child’s asthma (hereafter referred to collectively as caregivers) [6, 7]. The caregivers of children with asthma are tasked with administering medication, monitoring symptoms and triggers, and coordinating healthcare visits, which can lead to long-term stress and negatively impact work productivity and quality of life [8, 9]. Furthermore, the nocturnal symptoms of asthma can disrupt caregivers' sleep, resulting in daytime fatigue and impaired physical functioning, ultimately affecting their social and occupational relationships [8].

Previous systematic reviews have documented the significant impact of severe asthma on the QOL of both children and their caregivers [10, 11]. These studies have found that children with severe asthma and their caregivers often experience similar reductions in overall QOL, highlighting the broad and profound effects of the condition on entire families. In line with this, other studies have identified a significant correlation between improved asthma control in children and higher quality of life for their caregivers [12].

However, no previous reviews have comprehensively examined how the quality of life (QOL) of parents and caregivers varies depending on the severity and control of their child’s asthma. This is an important gap, as both asthma severity and asthma control are central to the management and prognosis of the disease, and they may differentially affect the caregiver experience.

Asthma severity and control are related but distinct concepts that may independently influence caregiver burden. Asthma severity refers to the intrinsic intensity of the disease, often determined at diagnosis or during clinical follow-up, and typically indicates how much treatment is required to achieve control [13–15]. In contrast, asthma control reflects the current effectiveness of treatment in managing symptoms, usually assessed during follow-up through symptom monitoring and response to medication [15, 16]. A child may have severe asthma that is well-controlled or mild asthma that is poorly controlled, and each of these scenarios may carry different implications for caregivers.

Understanding these distinctions is important because caregiver QOL may vary not only with the general presence of asthma but also with its severity and how well the disease is managed [5]. Yet many existing studies do not fully differentiate between levels of severity or control, nor do they explore how these levels might distinctly impact various QOL domains such as emotional well-being, functional capacity, and socio-occupational roles [10, 12].

Therefore, the main objective of this study is to systematically review the literature on the QOL of primary caregivers of children with asthma, focusing on comparing QOL across asthma severity and control categories. This includes comparisons between caregivers of children with severe versus non-severe asthma, controlled versus uncontrolled asthma, and children with asthma versus healthy children. Additionally, the study aims to clarify how each QOL domain is affected by severity and control, thereby providing more nuanced insights into the caregiver burden.

## Method

The systematic review is registered with PROSPERO (registration number: CRD42020169297) and follows the Preferred Reporting Items for Systematic Reviews and Meta-Analyses (PRISMA) guidelines.

### Search strategy

We conducted a comprehensive literature search across four databases: Web of Science, PubMed, Embase, and Scopus. The search used combinations of terms across four main areas: (1) asthma (e.g., “asthma*”, “wheez*”, “bronchospasm”), (2) child population (e.g., “child*”, “paediatric*”, “adolescen*”), (3) caregivers (e.g., “parent*”, “caregiver*”, “family*”), and (4) quality of life (e.g., “Quality of Life”, “HRQOL”, “PACQLQ”, “IFABI-R”). These terms were combined using Boolean operators (AND/OR). Search terms were adapted as needed for each database interface.

Searches were conducted in February 2020 and updated in October 2023. All studies published from inception to October 2023 were eligible. The search was limited to English-language publications.

### Study selection

The records of studies identified through the search strategy were first exported to EndNote, where duplicate entries were removed. Following this, three independent reviewers (MN, SR, and FT) conducted an initial screening of the titles and abstracts to determine studies’ eligibility for inclusion. Full-text articles of potentially eligible studies were then assessed against the predefined inclusion and exclusion criteria. Additionally, the reference lists of all eligible studies were reviewed to identify any other relevant articles that might have been missed. The study selection process at each stage was independently carried out by the three reviewers (MN, SR, and FT). Any disagreements that arose during the selection process were resolved through discussion. If a consensus could not be reached, a final decision was made after consulting the fourth reviewer (BM).

### Eligibility criteria

The inclusion criteria were defined as follows: (1) Study Design: We included cross-sectional studies, cohort studies, case–control studies, and randomised clinical trials. (2) Population: The studies included children between 5 and 18 years of age with clinically diagnosed asthma, encompassing all levels of asthma severity and control. Additionally, studies that compared children with asthma to a healthy control group were included. The studies needed to report data separately for QOL of children’s primary caregivers (who could be the one of the parents or any other specified caregiver responsible for child’s asthma management). (3) Setting: Studies conducted in any setting were considered. (4) Outcomes: The primary outcome was the overall caregiver/parent QOL scores, analysed across categories of asthma severity or control. The secondary outcomes involved the comparison of caregiver’s QOL domain scores (physical, emotional, and social) across different levels of asthma severity or control. (5) Publication Status: Only studies published in peer-reviewed journals were included, with conference papers, consensus statements, magazines, theses, book chapters, editorials, government documents, and patents excluded. (6) Language: Only studies fully published in English were considered for inclusion.

### Data extraction

A data extraction table was created using the Cochrane Effective Practice and Organisation of Care (EPOC) data collection form [17]. For each study, the following information was obtained: authors, publication year, study design, study population (number of children participating, children’s and caregivers’ age and gender, response rate, clinical characteristics including child’s asthma severity and control levels), participant selection criteria, outcomes (primary and secondary outcomes and measurement instruments), results, and other notable information such as sources of bias or conflicts of interest. Two reviewers (MN, SR) were initially involved in independently extracting data from the included studies using the pre-formatted data extraction table. A third reviewer (FT) was involved in selecting and extracting data for the search updates in 2023. Any discrepancies were resolved through discussion between the reviewers.

### Study quality assessment

The quality assessment was conducted using the modified Newcastle–Ottawa scale for cross-sectional studies [18]. Each study was evaluated on criteria including sample selection, representativeness, sample size justification, comparability between respondents and non-respondents, outcome assessment, quality assurance, statistical analysis, and response rate. The overall quality rating was calculated as the percentage of 'Yes' answers out of the total criteria. Studies were categorised as Good (> 75%), Fair (50–75%), or Poor (< 50%) based on this rating, with studies judged of good or fair quality included in the meta-analysis.

### Asthma severity and control

During data extraction, asthma severity and asthma control were treated as distinct constructs. Severity was recorded using standard clinical categories—intermittent, mild persistent, moderate persistent, and severe persistent—defined in guidelines such as those from the National Asthma Education and Prevention Program (NAEPP) and the Global Initiative for Asthma (GINA) [19]. Control was recorded using commonly applied categories—well-controlled, partly controlled, or poorly controlled—based on criteria from validated instruments, including the Asthma Control Questionnaire (ACQ) [20], Asthma Control Scoring System (ACSS) [21], and Childhood Asthma Control Test (C-ACT) [22, 23].

### Primary outcome

The primary outcome of this review was the caregiver’s standardised overall QOL score, reported separately for healthy control children and children with asthma, categorised by levels of asthma severity and control. We included both disease-specific (e.g., PACQLQ, IFABI-R) and general health-related QOL instruments (e.g., WHOQOL-BREF, EUROHIS-QOL-8) used in the included studies. Disease-specific tools are designed to be more sensitive to asthma-related changes in caregiver functioning, while general tools assess broader well-being but may be less responsive to condition-specific burden.

### Secondary outcomes

The secondary outcomes of the study were the caregivers’ standardised QOL scores for each given QOL domain (functional or activity limitations, emotional, and socio-occupational) among the caregivers of children categorised by varying levels of asthma severity and control. Several QOL questionnaires designed for parents and caregivers of children with asthma provide a comprehensive profile of the multi-dimensional construct of quality of life, in addition to a single overall index [11, 12].

### Statistical analysis

Caregiver QOL scores were summarised as means and standard deviation (SD). Other descriptive statistics like medians, interquartile ranges, or confidence intervals (CI) were extracted when means and SD were not provided.

We used a process to standardise the quality of life (QOL) scores on a 0 (worst) to 100 (best) points scale. First, we ensured that a higher score represents better QOL. This involved inverting the overall mean score if, on a particular instrument, a lower value represented a better QOL score. The inversion was done by subtracting the overall mean from the maximum possible score. Next, we standardised the inverted score to a 0–100 points scale by multiplying it by a factor that adjusts the maximum score to 100. This method ensures that the standardised scores are comparable across studies. For instance, if the original overall mean score is 2.5 on a scale where the best is 1.0 and the worst is 4.0, the standardised score would be 50 on a 100-point scale, with 100 indicating the best QOL. Standard errors (SE) and standard deviations (SD) were similarly standardised (see the supplemental materials on the methods for further details).

Next, we conducted a random-effects meta-analysis to combine study estimates and derive summary estimates of overall QOL and across individual QOL domains. The random-effects model was chosen because it assumes that QOL estimates can vary across studies due to real differences in QOL and sampling variability (chance) [24]. Heterogeneity in QOL estimates can arise from differences in study populations, such as age, gender, parenting status, severity scale used for assessing paediatric asthma, variations in follow-up length, and other study-specific factors.

The meta-analysis was performed using Stata 18, utilising the random-effects model to summarise the caregiver's overall QOL and domain-specific QOL across categories of child asthma severity or control. This model accounts for both within-study and between-study variability, acknowledging the likelihood of true differences in estimates across studies. By incorporating the random effects framework, we aimed to provide a more conservative estimate considering potential heterogeneity across studies. To ensure the robustness of our findings, we performed several checks for heterogeneity. We assessed the degree of heterogeneity between study estimates using the I-squared statistic [17]. Estimates for separate QOL questionnaires were also calculated and provided in the supplementary materials as a sensitivity analysis.

## Results

### Selected studies

**Fig. 1:**
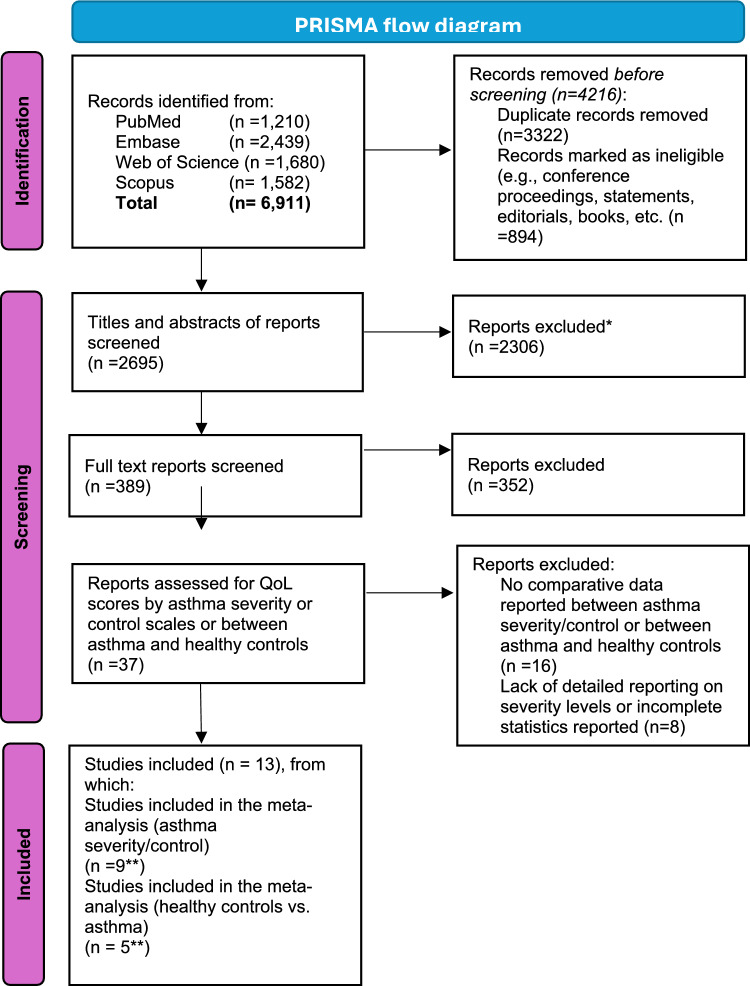
PRISMA flow diagram for the selection of studies. * Records excluded based on keywords search in all fields on asthma/wheeze and severity/control/management, and quality of life of parents/caregivers. ** One study [39] compared asthma patients to healthy controls as well as QoL scores by asthma severity levels. *Ref:* Page MJ, McKenzie JE, Bossuyt PM, Boutron I, Hoffmann TC, Mulrow CD, et al. The PRISMA 2020 statement: an updated guideline for reporting systematic reviews. BMJ 2021;372:n71. 10.1136/bmj.n71

The literature search conducted on online databases resulted in 6,911 records from PubMed (1,210 records), Embase (2,439 records), Web of Science (1,680 records), and Scopus (1,582 records). Before the screening, 4,216 records were removed, including 3,322 duplicates and 894 records marked as non-eligible (such as conference proceedings, statements, editorials, and books). After removing duplicates and ineligible records, 2,695 records remained for screening (Fig. 1). These records were first screened based on their titles and abstracts, focusing on keywords related to asthma, wheeze, severity, control, management, and the quality of life of caregivers/parents. This initial screening process aimed to identify studies that were potentially relevant to the research objectives before moving on to a full-text review for final inclusion. During this phase, 2,306 records were excluded, which left 389 reports to be screened based on their full text. Of the 389 studies screened, 352 were excluded for failing to meet the inclusion criteria. In total, 37 studies were considered for data extraction and screened for detailed QOL scores by asthma severity or control scales, or, for separately reporting asthma patients and healthy controls. Of these, 15 studies were excluded because they did not provide this level of detailed reporting. Additionally, one study did not report separate estimates by child’s asthma severity or control categories [25], two studies focused on very young children with wheeze (or a combination of wheeze and asthma) [26, 27], five studies lacked detailed reporting on severity levels or had incomplete statistics reported [28–32]. One study was excluded because it compared the QOL of caregivers of children with asthma to the general population, without specifying a healthy cohort [33]. Ultimately, 13 studies were included in the meta-analysis (Fig. 1). Nine studies [6, 7, 34–40] reported measures of caregivers’ QOL by asthma severity and control, while four [41–44] compared asthma patients to healthy controls. One study [39], compared both asthma patients to healthy controls and reported QOL scores by asthma severity levels (Table S2).

### Study quality assessment

The quality assessment of the 13 selected studies [6, 34–45] was assessed using the modified Newcastle–Ottawa scale for cross-sectional studies [18].

Out of these studies, 9 were rated as ‘Good’ [6, 35, 37, 38, 40–43, 45]. The remaining studies [34, 36, 39, 44] were rated as ‘Fair’. Common deficiencies included inadequate discussion of comparability between respondents and non-respondents and the lack of detailed sample size justification. No studies scored as ‘Poor’.

### Main characteristics of the selected studies

Table 1 summarises the selected studies, highlighting key aspects such as the study design, selection criteria, sample size, age and sex distribution, outcome measures, QOL dimensions assessed, and how asthma control or severity was categorised. The systematic review identified a wide range of studies conducted in Brazil [39, 43], the United States [6, 36, 37], Portugal [42, 45], India [34], Colombia [38], Spain [35] and the Netherlands [40, 41].

**Table 1:** Summary of the studies included in the meta-analysis

Study (year); Country	Study design; period of data collection	Selection criteria	Sample size (by asthma control/severity)	Children's Age (Mean ± SD) and Sex (Male/Female in %)	Parent's Age (Mean ± SD) and Sex (Male/Female in %)	Outcome measures	QoL dimensions	Asthma control/Severity
Comparison between severe/mild or moderate or controlled/uncontrolled asthma								
Battula et al. [34], India	Prospective cohort; 2017–2019	Children aged 7–17 newly diagnosed with asthma according to GINA guidelines, able to read and respond to the questionnaire in English	99 children and their caregivers	Mean Age: NA Sex: 64% male, 36% female	NA	Mini PAQLQ (Paediatric Asthma Quality of Life Questionnaire) and PACQLQ (Paediatric Asthma Caregiver’s Quality of Life Questionnaire) for QoL assessment, ACS (Asthma Clinical Severity) scoring, clinical examination, spirometry, BMI assessment	Mini PAQLQ (symptoms, activity limitation, emotional function) and PACQLQ (activity limitation, emotional function)	Asthma Clinical Severity Score (ACSS). Patients were classified as well-controlled (0.0 to 0.75), Partially controlled (0.76 to 1.5), and Poorly-controlled (> 1.5). Severity was measured at clinical diagnosis and control at 4 weeks follow-up
Roncada et al. [39], Brazil	Case–control; April 2015 to March 2016	Parents or caregivers of children with a diagnosis of asthma according to GINA criteria, as well as parents/caregivers of clinically healthy children and children with asthma in remission	101 parents/caregivers (50 asthma group, 51 control group)	NA	NA	WHOQOL-BREF questionnaire to assess quality of life in physical, psychological, social relations, and environmental domains as well as total score	Physical, Psychological, Social Relations, Environment domains and Total Score	Classified based on the GINA Classification of Asthma Severity diagnosis of asthma (persistent, mild, moderate, severe) or control (healthy or asthma in remission)
Cano-Garcinuno et al. [35], Spain	Prospective cohort	Main caregivers of 4–14-year-old children with asthma diagnosed by a doctor, having shown symptoms within the previous 12 months	462 caregivers	Mean Age: NA Sex: 63.4% male, 36.6% female	NA	Family Impact of Childhood Bronchial Asthma-Revised (IFABI-R) for QoL, PSI for caregivers' perception of asthma symptoms, ‘‘family APGAR’’ for family functioning, and clinical assessment for asthma control	IFABI-R: Functional, Emotional, and Socio-occupational dimensions	Asthma control was assessed by an experienced paediatrician based on based on clinical criteria (Third National Asthma Expert Panel Report, NAEPP-3) and was classified into three levels: good, partial, and poorly controlled
Everhart et al. [46], USA	Cross-sectional	7–12 years, diagnosed with asthma, caregiver identified as African American/Black, caregiver in charge of daily asthma care	55 dyads	Mean Age: 9.67 ± 1.50 Sex: NA	NA	Caregiver QOL, asthma control, ED visits due to asthma	PACQLQ (including activity limitation and emotional function subscales)	Assessed via Asthma Control Test (ACT) and reported ED visits
Rodriguez-Martinez et al. [38], Colombia	Prospective cohort validation study	Asthmatic children aged 7–17 years and their parents/primary caregivers attended a baseline and a follow-up visit 2–6 weeks later. Patients with other significant chronic disorders or congenital abnormalities were excluded	118 patients 66 (55.9%) had controlled asthma, 10 (8.5%) partly controlled asthma, and 42 (35.6%) uncontrolled asthma	Mean Age: 10.19 (est) Sex: 53.4% male, 46.6% female	NA	PACQLQ scores, asthma control	Overall QoL, activity limitations and emotional function domains as measured by the PACQLQ	Asthma control was assessed based on the GINA guideline recommendations in three categories (controlled, partly controlled and uncontrolled)
Silva et al. [7], Portugal	Cross-sectional; September 2010–February 2012	8–18 years of age, diagnosed with asthma by a physician	180 dyads of children and parents	Mean Age: 11.98 ± 2.57 Sex: 64.4% male, 35.6% female	Mean Age: 41.03 ± 5.77	Parents' and children's QoL, relationship burden, objective burden, subjective burden, and uplifts	Parents' QoL measured by EUROHIS-QOL 8-item index; Children's QoL measured by KIDSCREEN-10 Index	Asthma severity according to GINA and categorised into four categories: intermittent, mild persistent, moderate persistent, severe persistent; Control levels: controlled, partly controlled, uncontrolled
Okelo et al. [37],United States	Cross-sectional study; July 2007–September 2010	Children with self-reported doctor-diagnosed asthma, 21 years of age or younger, accompanied by a caregiver, English or Spanish speakers	317 children (265 English-speaking, 52 Spanish-speaking)	Mean Age: 7.94 (est); 8.2 years English-speaking; 6.6 years Spanish-speaking Sex: 58% male, 42% female	NA	Asthma Control Test (ACT)/Childhood Asthma Control Test (C-ACT), Paediatric Asthma Caregiver's Quality of Life Questionnaire (PACQLQ), spirometry	Assessed impact on activity limitation and emotional function	Asthma control assessed based on PACCI Control domain and ACT for children 4–11 years. Two control categories were used (‘controlled’ and ‘not controlled’). ‘Intermittent’ symptoms considered ‘controlled,’ whereas ‘persistent’ symptoms considered ‘not controlled.’
van Bergen et al. [40]; The Netherlands	Cross-sectional	Children 4 to 18 years of age with a physician’s diagnosis of atopic asthma based on clinical symptoms, FEV1 bronchodilator response of > 9% of predicted, and/or airway hyperresponsiveness and/or FENO > 25 ppb. Patients had been using inhaled corticosteroids for at least 3 months before inclusion and had Internet access at home	228 children 4–11 y old: (n = 151) 12–18 y old (n = 77)	Mean Age: 10.5 ± 3.0 Sex: 67% male, 33% female	NA	Paediatric Asthma Caregivers Quality of Life Questionnaire (PACQLQ) for children under 12 years and Paediatric Asthma Quality of Life Questionnaire (PAQLQ) for children 12 years and older	Domains related to emotions, activity, and symptoms equally weighted for PAQLQ and how caregivers are limited in their own quality of life by their child’s asthma for PACQLQ. Maximal score in both tests is 7 indicating optimal quality of life​​	Based on the diary data, the patients were categorised as having either well-controlled, partly controlled, or uncontrolled asthma according to GINA guidelines including FEV1​
Cerdan et al. [6], USA	Cross-sectional; August 2008–February 2009	Parents of children aged 7–17 with medical diagnoses of mild intermittent to severe persistent asthma	101 parents	Mean Age: NA Sex: 55.4% male, 44.6% female	NA	PACQLQ, Asthma Severity Questionnaire, Sociodemographic Factors Questionnaire	Activity limitation, Emotional function (PACQLQ)	Own questionnaire based on clinical criteria (Third National Asthma Expert Panel Report, NAEPP-3), medication, spirometry readings and also days lost off school. Four levels: mild intermittent, mild persistent, moderate persistent, and severe persistent asthma
Comparisons between asthma and healthy controls								
Roncada et al. [39], Brazil	(see above)							
Roncada et al. [43], Brazil	Cross-sectional	Parents of asthmatic and healthy children aged between 8 and 16. Exclusion criteria included parents with asthma or other chronic diseases that could interfere with the study results	Parents of asthmatic children (n = 54), Parents of healthy children (n = 108)	Mean Age: NA Sex: NA	Mean Age: 43.8 ± 13.6 Sex: NA	WHOQOL-BREF to assess QOL; internal consistency measured using Cronbach’s alpha (˛C) and intraclass correlation coefficient (ICC)	Physical, Psychological, Social Relationships, and Environment domains evaluated by WHOQOL-BREF	Asthma diagnosis for the children. No severity/control levels reported
Moreira et al. [42], Portugal	Cross-sectional; March 2009–December 2012	Children/adolescents aged 8–18 with chronic conditions (diabetes, asthma, epilepsy, CP, obesity) or healthy controls	Diabetes (n = 85) Asthma (n = 308) Epilepsy (n = 68) CP (n = 94) Obesity (n = 110) Healthy (n = 299)	Mean Age: Cases: 12.24 ± 2.64; Controls: 11.75 ± 3.25 Sex: 46.2% male, 53.2% female	Mean Age: Cases: 41.27 ± 5.82; Controls: 41.72 ± 5.54 Sex: NA	KIDSCREEN-10 index for children’s QoL, Strengths and Difficulties Questionnaire for psychological adjustment, EUROHIS-QOL-8 for parents' QoL​​	General QoL, Internalizing problems, Externalizing problems for children; Parents’ QoL	Following the GINA guidelines, asthma severity was classified by clinicians into 4 categories: intermittent, mild persistent, moderate persistent, and severe persistent
Hatzmann et al. [41], The Netherlands	Retrospective study (cross-sectional data for QoL); January 2006–September 2007	Parents of children with chronic conditions (10 diagnosis groups, children aged 1–19 years, diagnosed > 1 year ago, living at home) and parents of schoolchildren	Cases: 87 parents of children/adolescents with asthma Controls: 425 parents of healthy children/adolescents	Mean Age: 10.7 ± 4.3 Sex: NA	Mean Age: Cases 42.2 ± 6.7 Controls: 43.7 ± 5.5 Sex: 13.8% male, 86.2% female	TNO-AZL Questionnaire for Adult’s Health Related Quality of Life (TAAQOL)	HRQOL measures gross and fine motor function, cognitive functioning, sleep, pain, social functioning, daily activities, sexuality, vitality, positive and depressive emotions, aggressiveness​​	Asthma was defined as one of the selected chronic conditions in children. No severity/control levels reported
Van Gent et al. [44], Netherlands	Cross-sectional; September 2002–April 2005	Children aged 7–10 years in primary schools and their parents. Children were identified as ‘diagnosed asthma’ (DA), ‘undiagnosed asthma’ (UDA), or ‘healthy controls’ (HC)	UDA (n = 130), DA (n = 81), HC (n = 202)	Mean Age: 9.4 ± 0.7 Sex: 58% male, 42% female	NA	Paediatric Asthma Quality of Life Questionnaire (PAQLQ) for children and Paediatric Asthma Caregiver’s Quality of Life Questionnaire (PACQLQ) for caregivers; school absence due to respiratory symptoms	Emotions and Activity domains for caregivers	Classified based on results from questionnaires, airway reversibility, and bronchial hyperresponsiveness (BHR)

*PAQLQ* Pediatric Asthma Quality of Life Questionnaire, *PACQLQ* Pediatric Asthma Caregiver’s Quality of Life Questionnaire, *IFABI-R* Family Impact of Childhood Bronchial Asthma-Revised, *WHOQOL-BREF* The World Health Organization Quality of Life Brief Version, *TNO-AZL* Netherlands Organization for Applied Scientific Research/Academic Hospital Leiden Center Children's Quality of Life, *ACT* Asthma Control Test, *C-ACT* Childhood Asthma Control Test

#### Summary of study designs and populations

The selected studies used a variety of designs, including prospective cohort studies, cross-sectional studies and case–control studies. The studies included children aged 4 to 18, with the mean age varying slightly from 8–9 [36, 37, 44, 47] to 11–12 [42, 45] between studies. There were more male participants across all studies, with percentages ranging from 53.4 to 64.4%. The mean age of parents and caregivers also varied across studies, with reports ranging from mid-30 s [35] to mid-40 s [45].

#### Quality of life measures and outcomes

A number of validated questionnaires were used to assess QOL in the selected studies. The most frequently used tool was the Pediatric Asthma Caregiver’s Quality of Life Questionnaire (PACQLQ), employed in seven studies [6, 34, 36–38, 40, 44]. The PACQLQ focuses on activity limitations and emotional function, providing a consistent framework for comparing how asthma affects daily life and emotional well-being across diverse populations [48].

Similarly, the Family Impact of Childhood Bronchial Asthma-Revised (IFABI-R) used in one of the selected studies [35] offers a comprehensive view of the multidimensional impact of asthma on family dynamics by assessing functional, emotional, and socio-occupational dimensions [27].

Other QOL questionnaires used were the World Health Organization Quality of Life Brief Version (WHOQOL-BREF) utilised in two selected studies [39], and EUROHIS-QOL-8 (an adaptation of WHOQOL-BREF) used in two further studies [42, 45]. These later tools provided a broader assessment of overall QOL, including physical, psychological, social, and environmental domains of children’s asthma on parents’ QOL [49–51] (Table S2).

#### Asthma control and severity classification

Asthma control and severity in the selected studies were assessed using a number of standardised tools. Seven of the 14 studies included [34–38, 40, 45] used the Childhood Asthma Control Test (C-ACT) to classify asthma into control categories such as well-controlled, partly controlled, and poorly controlled, based on symptoms, rescue medication use, and the impact on daily activities. The C-ACT is a widely validated tool providing a consistent framework for comparing asthma control across different populations [22, 23].

Similarly, asthma severity classifications in eight studies [6, 34, 35, 37–40, 45] were based on established guidelines, such as the Global Initiative for Asthma (GINA) [52] or the National Asthma Education and Prevention Program (NAEPP-3) [19]. These guidelines help categorise asthma into mild intermittent, mild persistent, moderate persistent, and severe persistent levels, facilitating uniform comparisons of the impact of severity across studies. Consistent use of these standardised tools across studies strengthens the validity of comparisons and meta-analyses of QOL outcomes related to asthma control and severity.

### Meta-analysis of QOL scores

#### Overall QOL scores

**Fig. 2:**
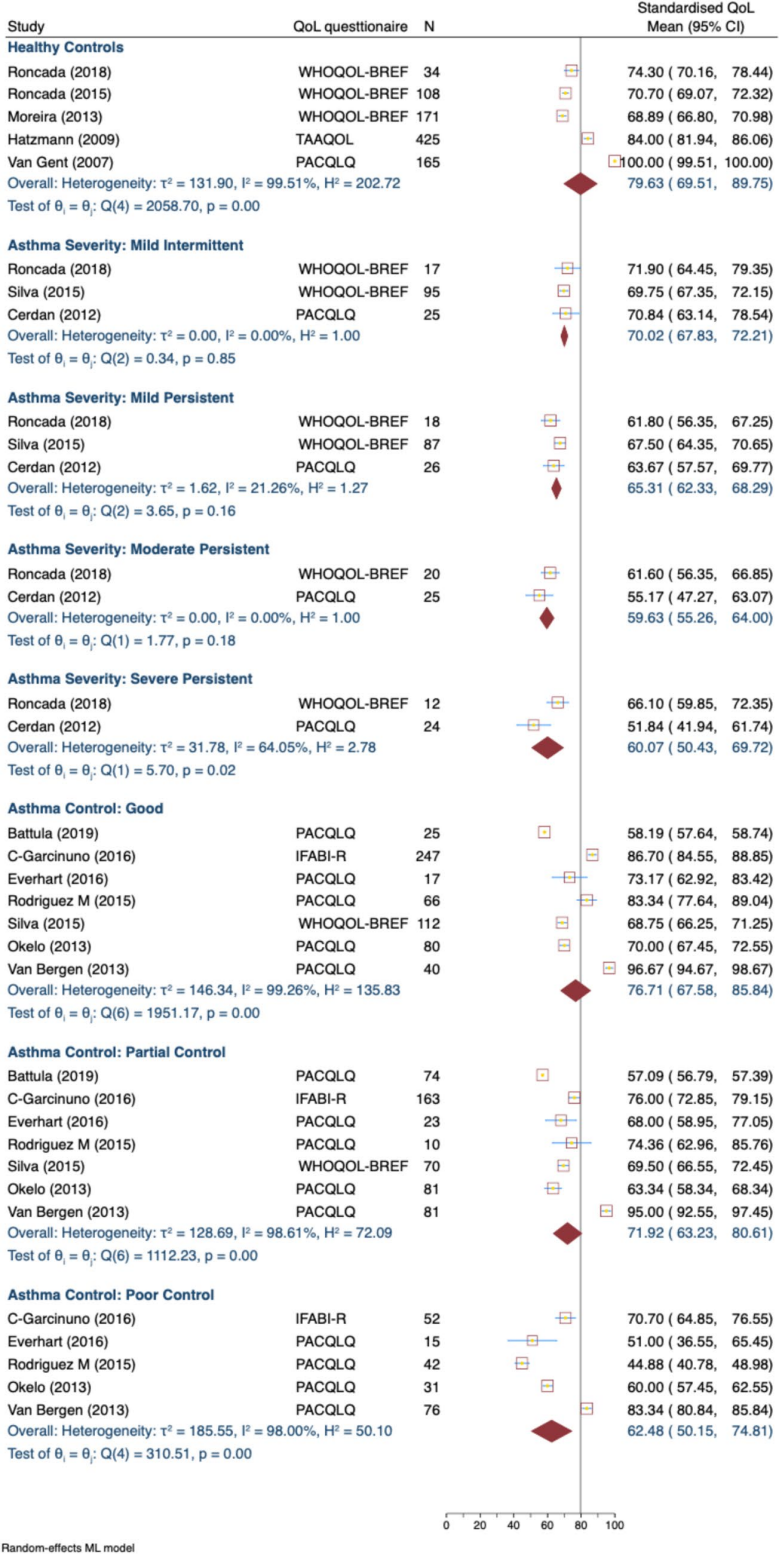
Standardised overall caregiver QoL score, by asthma severity and control levels. *WHOQOL-BREF* The World Health Organization Quality of Life Brief Version, *PACQLQ* Pediatric Asthma Caregiver’s Quality of Life Questionnaire, *IFABI-R* Family Impact of Childhood Bronchial Asthma-Revised, *TAAQOL* Netherlands Organization for Applied Scientific Research/Academic Hospital Leiden Center Children's Quality of Life

In terms of overall QOL scores, caregivers of healthy controls (Fig. 2) had the highest QOL scores, with an overall estimate of 79.63 (95% CI: 69.51, 89.75; 5 studies) [39, 41–44]. In contrast, caregivers of children with asthma demonstrated a pattern of lower QOL scores, particularly if the child’s asthma was classified as more severe. For example, caregivers of children with moderate persistent asthma had a score of 59.63 (95% CI: 55.26, 64.00; 2 studies [6, 45]), and those with severe persistent asthma had a score of 60.07 (95% CI: 50.43, 69.72; 2 studies [6, 39]).

In the comparisons of overall QOL by asthma control levels, caregivers of children with good asthma control reported a higher QOL score of 76.71 (95% CI: 67.58, 85.84; 4 studies [35, 38, 40, 46]), while those with poorly controlled asthma had a lower score of 62.48 (95% CI: 50.15, 74.81; 4 studies [35, 38, 40, 46]). Heterogeneity was significantly higher, especially for the groups of healthy controls (I^2^ = 99.51%), good asthma control (I^2^ = 99.26%), partial asthma control (I^2^ = 98.61%), and poorly controlled asthma (I^2^ = 98.00%).

#### Activity QOL scores

**Fig. 3:**
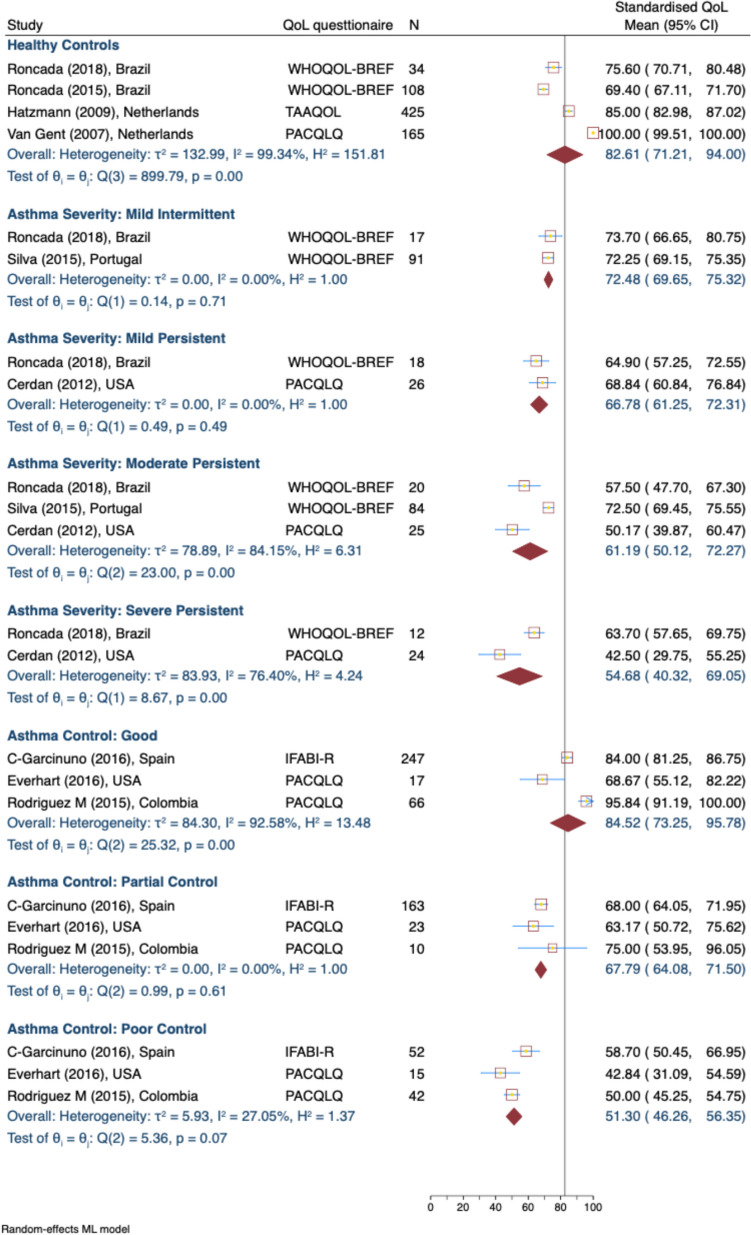
Standardised caregiver activity domain of QOL score, by asthma severity and control levels. *WHOQOL-BREF* The World Health Organization Quality of Life Brief Version, *PACQLQ* Pediatric Asthma Caregiver’s Quality of Life Questionnaire, *IFABI-R* Family Impact of Childhood Bronchial Asthma-Revised, *TAAQOL* Netherlands Organization for Applied Scientific Research/Academic Hospital Leiden Center Children's Quality of Life

In the QOL activity domain (Fig. 3), caregivers of healthy children again reported the highest scores at 82.61 (95% CI: 71.21, 94.00; 4 studies [39, 41, 43, 44]). Caregivers of children with severe persistent asthma reported much lower activity scores of 54.68 (95% CI: 40.32, 69.05; 2 studies [6, 45]. Similarly, asthma control impacted activity scores: caregivers of children with good control of asthma had higher scores of 84.52 (95% CI: 73.25, 95.78; 3 studies [35, 38, 46] compared to those with poor asthma control of 51.30 (95% CI: 46.26, 56.35; 3 studies [35, 38, 46]). Heterogeneity was high, particularly for the healthy control group (I^2^ = 99.34%), moderate persistent (I^2^ = 84.15%) and severe persistent asthma (I^2^ = 76.40%) and good asthma control (I^2^ = 92.58%).

#### Emotional QOL scores

**Fig. 4:**
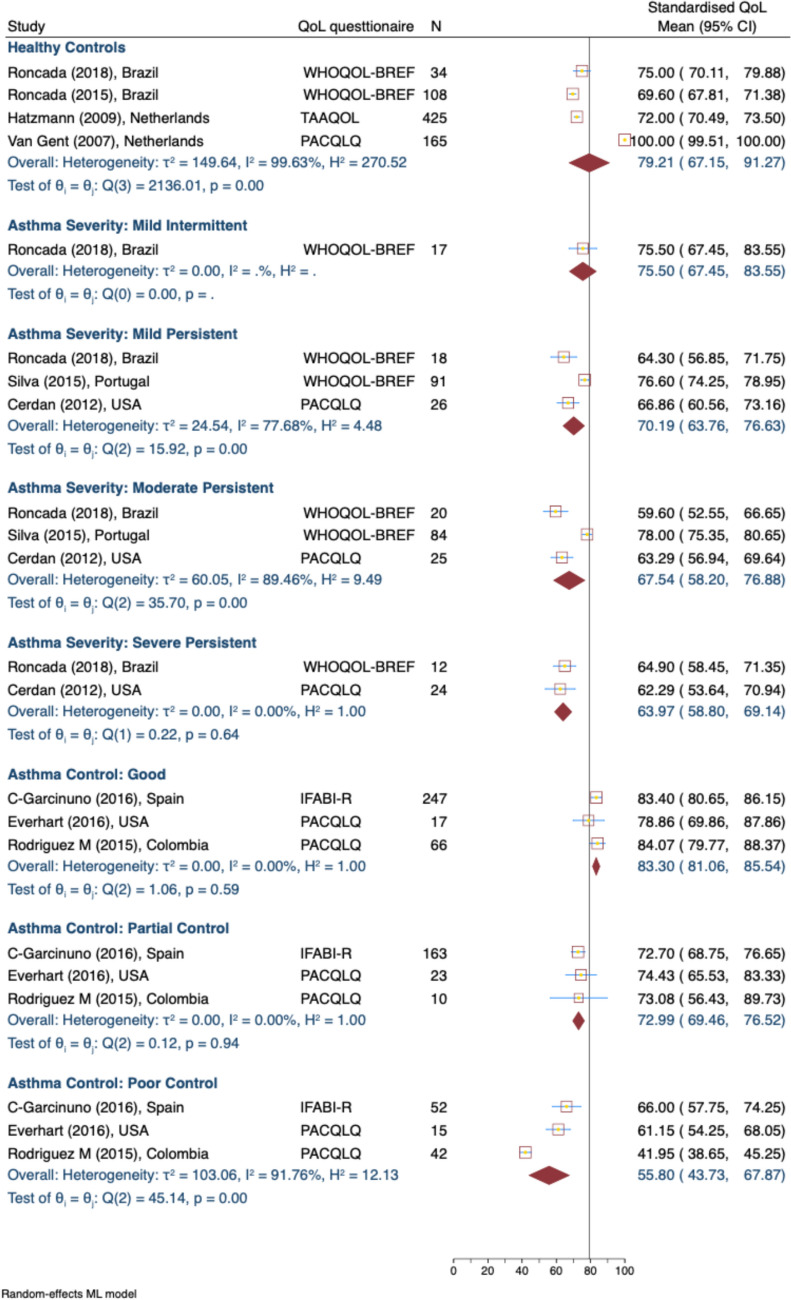
Standardised Caregiver Emotional Domain of QOL Score, by Asthma Severity and Control Levels. *WHOQOL-BREF* The World Health Organization Quality of Life Brief Version, *PACQLQ* Pediatric Asthma Caregiver’s Quality of Life Questionnaire, *IFABI-R* Family Impact of Childhood Bronchial Asthma-Revised, *TAAQOL* Netherlands Organization for Applied Scientific Research/Academic Hospital Leiden Center Children's Quality of Life

Caregivers of healthy children had high scores of 79.21 (95% CI: 67.15, 91.27; 4 studies [39, 43, 44, 47]) for the emotional QOL domain (Fig. 4), while those with children suffering from severe persistent asthma reported lower scores of 63.97 (95% CI: 58.80, 69.14; 2 studies [6, 45]. Similarly, good control of asthma corresponded to higher emotional QOL scores of 83.30 (95% CI: 81.06, 85.54; 3 studies [35, 36, 38] while poor control was associated with significantly lower scores of 55.80 (95% CI: 43.73, 67.87; 3 studies [35, 36, 38]). Heterogeneity was again substantial across most domains.

#### Socio-occupational QOL scores

**Fig. 5:**
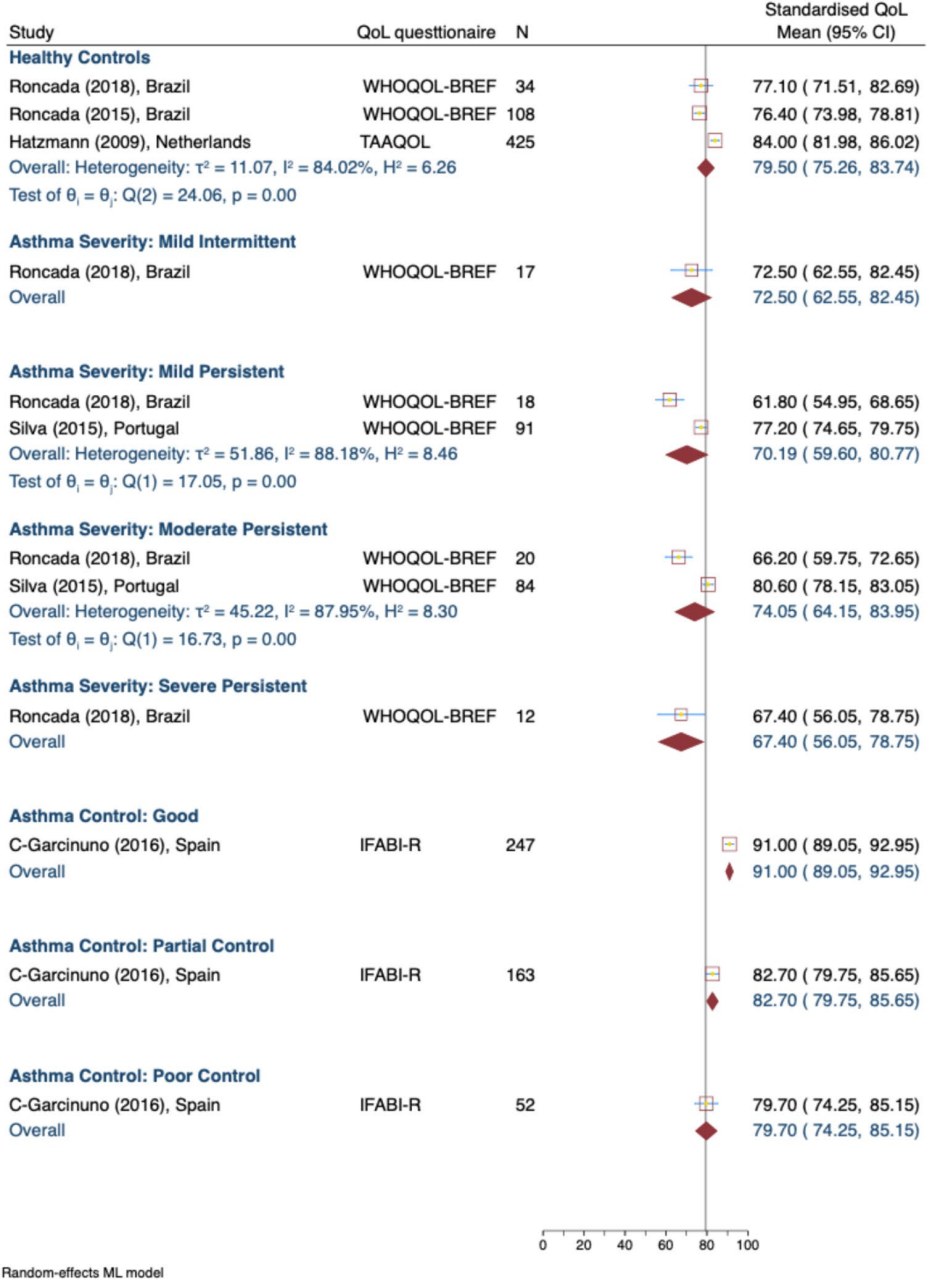
Standardised caregiver socio-occupational domain of QOL score, by asthma severity and control levels. *WHOQOL-BREF* The World Health Organization Quality of Life Brief Version, *IFABI-R* Family Impact of Childhood Bronchial Asthma-Revised

Due to limited data reported in the selected studies, a meta-analysis could not be conducted for most of the subgroups of asthma control or severity levels of the socio-occupational QOL domain (Fig. 5). The available data show again that caregivers of healthy children reported the highest scores of 79.50 (95% CI: 75.26, 83.74; 3 studies [39, 41, 43]). Nevertheless, higher heterogeneity (I^2^ = 84.02%) was observed, reflecting variability across studies.

## Discussion

This systematic review synthesises the available evidence on the QOL of the primary caregivers of children with asthma, focusing on the differences between severe versus non-severe asthma, controlled versus uncontrolled asthma, and comparing these scores to the QOL of caregivers of children without asthma. We identified three studies comparing QOL across severity levels [6, 39, 45], seven studies comparing QOL across asthma control levels [34, 35, 37, 38, 40, 45, 46], and five studies comparing healthy controls against children with asthma [39, 41–44]. We show here that the overall QOL scores were reportedly highest among caregivers of healthy children and progressively lower in those caring for children with more severe or poorly controlled asthma.

### QOL patterns across domains

Caregivers of children with severe persistent asthma reported notably low QOL scores in the activity domain [6, 45]. These limitations were even more pronounced among caregivers of children with poorly controlled asthma, who consistently reported the lowest activity scores across studies [35, 38, 46]. Activity-related burdens stem from responsibilities such as frequent symptom monitoring, managing acute exacerbations, and attending medical appointments. These demands often disrupt daily routines, restrict leisure and social activities, and interfere with employment [53, 54]. These burdens are heightened among caregivers of children with poorly controlled asthma, where unpredictable and frequent symptom episodes intensify disruptions to family and social life [55].

Emotional burden was a notable theme in the reviewed studies, with caregivers of children with poorly controlled asthma often reporting the lowest quality of life (QOL) scores in the emotional domain [35, 38, 46]. Lower emotional QOL was also observed in caregivers of children with moderate and severe persistent asthma [6, 45]. These findings align with previous research [11, 55, 56] and reflect the psychological stress of caregiving, including continuous symptom monitoring, fear of exacerbations, and responsibility for medication adherence. Our meta-analysis suggests that the emotional well-being of these caregivers is comparable to that of parents of paediatric oncology patients, as reported in prior studies [57–59], emphasising the significant psychological burden they face.

Although assessed less frequently, the socio-occupational domain emerged as a critical but underexplored aspect of caregiver QOL. Studies that examined this domain found lower scores among caregivers of children with severe or poorly controlled asthma [41, 55]. Lower caregiver socio-occupational QOL scores have been linked to reduced work productivity, disrupted career trajectories, and strained household and social roles—factors that may contribute to challenges and significant implications for healthcare access, family stability, and long-term wellbeing [55].

### Distinguishing between asthma severity and control

Our review underscores the importance of distinguishing between asthma severity and asthma control. It expands on previous systematic reviews that focused on QOL in caregivers of children with asthma [7, 11, 12], typically examining control or severity alone. This dual perspective offers a more comprehensive understanding of how each construct contributes uniquely and jointly to the caregiver experience across various QOL domains, and compared to caregivers of healthy children.

Asthma control and asthma severity are interpreted differently in both clinical and research contexts. Control reflects the current status of the disease—how well symptoms and risks are managed with treatment—while severity refers to the intrinsic intensity of the disease, assessed retrospectively by the level of treatment needed to achieve that control [52], #5052}. According to the GINA’s Global Strategy for Asthma Management and Prevention [52], a person may have severe asthma even if symptoms are currently well controlled, if high-intensity treatment is required to maintain that state. In this context, asthma severity reflects a more stable, underlying disease classification and is typically stable over time, requiring consistent treatment regardless of current symptoms. In contrast, asthma control can vary with day-to-day medication adherence, environmental exposures, and access to care. This distinction is important for understanding both short-term burden and long-term treatment complexity in asthma care [15].

Our findings suggest that caregiver QOL—particularly in emotional and activity-related domains—is more sensitive to the levels of asthma control than severity classifications. This aligns with the meta-analysis by Costa et al. [12], which found a strong and statistically significant association between asthma control scores and caregiver QOL (R^2^ = 0.82; p < 0.001).

However, asthma severity still plays a meaningful role. Caregivers of children with severe persistent asthma often manage sustained clinical demands, such as regular specialist care, complex treatment regimens, and increased risk of exacerbations [10]. These burdens may continue even when day-to-day symptoms appear controlled. Recognising this distinction is important in designing interventions that address both the episodic strain associated with poor control and the chronic caregiving stress linked to severe asthma.

### Sources of heterogeneity in caregiver QOL estimates

We observed substantial heterogeneity in caregiver QOL estimates across domains, which appears to stem from several sources. One key factor was variation in the tools used to measure both QOL and asthma classification. Studies employed a range of QOL instruments (e.g., PACQLQ, IFABI-R, WHOQOL-BREF) and used different definitions of asthma severity and control (e.g., GINA, NAEPP-3, ACS, ACT-C). To allow comparison, we standardised QOL scores to a 0–100 scale; however, this assumes that all tools are equally sensitive to changes in caregiver experience, a limitation worth noting. Disease-specific measures like PACQLQ were generally better at detecting subtle changes in emotional functioning and daily life than broader tools such as WHOQOL-BREF, which may miss asthma-specific challenges [48, 60–63].

Another contributing factor was the variation in clinical settings. Some studies were conducted in general practice, while others were based in specialist asthma centres, which may offer more structured support, better education, and access to multidisciplinary care. These differences in service delivery may shape caregiver experience, but such contextual details were often not reported in enough detail to explore their influence fully.

These observations highlight the value of using standardised, well-validated tools to assess QOL and asthma status. Greater alignment in measurement tools and classification methods would enhance the comparability of findings and strengthen the evidence base for understanding caregiver burden [64, 65].

### Caregiver–child dynamics

Caregiver and child QOL are closely linked and likely bidirectional. Although not directly in the scope of our review, one of the selected studies [38] reported a strong correlation between the two. At the same time, broader literature suggests that caregiver stress, sleep loss, and poor coping can impair asthma management and worsen child outcomes [66]. Emotional distress may reduce medication adherence, delay responses to symptoms, and increase family conflict, which in turn, may further exacerbate asthma in children [66, 67].

However, establishing a direct causal link—specifically, that childhood asthma causes decreased parental QOL—is challenging due to reverse causation and confounding variables, such as pre-existing stress, socioeconomic status, and environmental influences. While observational studies provide useful insights, they often fall short of confirming causation. In contrast, quasi-experimental and longitudinal studies may offer stronger evidence by controlling for these variables.

### Clinical and policy implications

Our findings emphasise the need for targeted interventions to reduce asthma severity and improve symptom control. Patient education has the potential to be a cornerstone of clinical practice [68], with clinicians actively engaging families in understanding the link between asthma management and improved QOL. Educating families on the importance of effective asthma control can enhance caregivers' QOL and daily functioning [69]. Identifying specific QOL challenges allows healthcare providers to allocate resources more effectively, offering holistic care. Psychosocial interventions, such as counselling, cognitive-behavioural therapies, and social support, can also reduce parental burden and promote overall well-being [70], ultimately leading to better health outcomes for children.

Although caregiver education and self-management were not directly assessed in this review, evidence from prior studies suggests that structured support, including asthma action plans, problem-solving training, and peer support, can reduce caregiver burden and improve both QOL and asthma outcomes [7, 45, 68, 71–73]. Future trials need to incorporate caregiver-focused components and examine them as mediators of child outcomes.

### Study limitations

This review has several limitations. First, only one study [39] directly compared asthma severity or control categories to a healthy control group. We added other studies with healthy controls for context, but the lack of direct comparisons limited interpretation. This underscores the need for future research to include healthy control groups to establish more robust reference points. Second, most of our included studies used a convenience sampling method, leading to under- or overrepresentation of specific groups within the sample and, therefore, increasing the susceptibility to selection bias [74]. Third, differences between children, in addition to their level of asthma severity or control, may have impacted the quality of life of their caregivers; further studies should aim to better control for such differences. In addition, while our study highlights the differences between severity and control scales, it is important to recognise their interrelation. Asthma control can influence the perceived severity, and both scales often use similar indicators, such as symptom frequency and medication use, to assess outcomes. This overlap makes it challenging to fully isolate their individual impacts on QOL, adding complexity to distinguishing between the effects of severity and control.

And, lastly, our search strategy was limited to studies published in the English language only, and the “grey literature” was not considered, which may have contributed to publication bias.

## Conclusions

This review emphasises the significant burden that caring for children with asthma may have on the QOL of their parents and caregivers, especially when the asthma is severe or poorly controlled. Our findings show that primary caregivers of children with more severe asthma or poor asthma control report notably lower QOL scores in activity limitations and emotional well-being compared to those caring for children with milder or well-controlled asthma. These results highlight the need for targeted interventions to reduce caregiver stress and burden. It is important to prioritise psychosocial support, including counselling, cognitive-behavioural therapies, and social support systems, to enhance the overall well-being of caregivers. By addressing these needs, healthcare providers can improve not only the QOL of parents but also the management and health outcomes for children with asthma. Effective asthma management strategies and supportive resources are crucial not only for achieving and maintaining good asthma control but also for reducing the sustained burden associated with more severe forms of the disease. Together, these efforts help minimise caregiver stress and support comprehensive care for both children and their families.

## Supplementary Information

Below is the link to the electronic supplementary material.

Supplementary file1 (DOCX 432 kb)

## Data Availability

This study is based on a systematic review of published literature. No new data were generated or analysed during this study. All data supporting the findings are derived from previously published studies, which are cited in the manuscript. Full details of the search strategy and study selection process are provided in the Methods.
