# Supplementary material for: Quality of life of primary caregivers by severity and control of children’s asthma: a systematic review and meta-analysis
Source: Qual Life Res. 2025 Aug 27;34(12):3425–45. doi: 10.1007/s11136-025-04042-6 (PMC12689705; doi:10.1007/s11136-025-04042-6)
Supplement: Supplementary file 1 — Supplementary file1 (DOCX 432 kb) [file 11136_2025_4042_MOESM1_ESM.docx]

***Supplemental material***

**Table S1: Search strategy for all electronic databases**

| Number | Search statement |
| --- | --- |
| 1 | “asthma*" OR "bronchial asthma" OR “bronchial constrict” OR “bronchoconstrict" OR “bronchial spas*” OR “bronchospasm” OR “bronchial hypersensitivity” OR “respiratory hypersensitivity” OR “wheez*” |
| 2 | “child*” OR “children*” OR “paediatric*” OR “pediatric*” OR “adolescen*” OR “teenager*” OR “young*” OR “youth” |
| 3 | “parent*” OR “mother*” OR “father*” OR “caregiver*” OR “carer*” OR “family*” OR “guardian*” |
| 4 | “Quality of Life” OR “Health-Related Quality of Life” OR “QOL” OR “HRQOL” OR “PACQLQ” OR “IFABI-R” |
| 5 | 1 AND 2 AND 3 AND 4 |

**Table S2. Selected studies and the QoL domains covered**

| **No** | **Study, Year, Country** | **Overall** | **Activity** | **Emotional** | **Socio-occupational** |
| --- | --- | --- | --- | --- | --- |
| **Comparison between severe/mild or moderate or controlled/uncontrolled asthma** | | | | | |
| 1 | Battula et al. (2020); India | ✓ |  |  |  |
| 2 | Roncada et al. (2018); Brazil | ✓ | ✓ | ✓ | ✓ |
| 3 | Cano-Garcinuno et al. (2016); Spain | ✓ | ✓ | ✓ | ✓ |
| 4 | Everhart et al. (2016); USA | ✓ |  |  |  |
| 5 | Rodriguez-Martinez et al. (2015); Colombia | ✓ | ✓ | ✓ |  |
| 6 | Silva et al. (2015b); Portugal | ✓ | ✓ | ✓ | ✓ |
| 7 | Okelo et al., 2013; United States | ✓ |  |  |  |
| 8 | van Bergen et al. (2013); The Netherlands | ✓ |  |  |  |
| 9 | Cerdan et al. (2012); USA | ✓ | ✓ | ✓ |  |
| **Comparison between asthma and healthy controls** | | | | | |
| 1 | Roncada et al. (2018); Brazil | ✓ | ✓ | ✓ | ✓ |
| 2 | Roncada et al. (2015); Brazil | ✓ | ✓ | ✓ | ✓ |
| 3 | Moreira et al. (2013); Portugal | ✓ |  |  |  |
| 4 | Hatzmann et al. (2008); The Netherlands |  | ✓ | ✓ | ✓ |
| 5 | Van Gent et al. (2007); Netherlands | ✓ |  |  |  |

**Supplemental methods**

***Calculating the CI Interval from the sample mean, SD and sample number***

To calculate the 95% confidence interval (CI) for the mean of a normally distributed population when the standard deviation (SD) is known, we have used the following formula:

$$CI=\bar{x}\pm Z\times\left( \frac{SD}{\sqrt{n}} \right)$$

Where:

- $\bar{x}$ is the sample mean

- $Z$ is the Z-score from the standard normal distribution for the desired confidence level (for the 95% CI, the Z is typically 1.96)

-$\mathrm{SD}$ is the standard deviation of the sample

- $n$ is the sample size

***Inverting and/or Standardising QoL scores across QoL scales***

Some of the selected studies have included QoL scores, where scoring scale ranges from lowest value=best to highest value= worst QoL. In addition the range of the scale could differ from 0-100 scale. For instance, IFABI-R score ranges between 1 (best) and 4 (worst) quality of life.

Suppose we only have the overall mean IFABI-R score and we wish to standardise this composite score to a 0 (worst)-100 (best) QOL scale. In that case, we follow the following steps:

1. Invert the overall mean.

First, since a lower score is better, we must invert the scale to align with desired ordering across the scale of a higher score is better. In the case of IFABI-R scores, this means extracting the score values from the maximum value of 4 so the inverted score range is 0 to 3 with inverted mean score calculated as:

$\text{Inverted Mean}=4-\text{Overall Mean}$

1. We standardise the inverted score scale ranging from 0 to 3 to a 100-point scale by multiplying by 100/3 since the maximum of 3 now corresponds to the maximum of 100. Therefore to standardise the inverted mean to 0-100 scale

$$\text{Standardised }\text{Inverted Mean }\text{Score}=\text{Inverted Mean}\times\frac{100}{3}$$

This gives us a standardised score out of 100, where a higher number indicates a better quality of life. As an example, let's say that the overall mean score of IFABI-R scores is 2.5 across the four domains. The standardised score on a 100-point scale would be:

$$InvertedMean=4-2.5=1.5$$

$$StandardisedScore=1.5\times\frac{100}{3}=50$$

So, the standardised mean score out of 100 would be 50.

***Calculating the Inverted and/or Standardised SD:***

The steps we follow to standardise the standard deviation of the scores are as follows:

1. Note that although the mean was inverted, the standard deviation (SD) of the scores does need to be modified. The SD remains the same because the spread (or variability) does not change when the scores are inverted.
2. Standardise SD.

To standardise the SD to a 100-point Scale, however, the standard deviation must also be multiplied by the same factor used to standardise the mean. In the case of IFABI-R, for example, the scaling factor is:

$$\text{Standardised SD}=\text{Original SD}\times\frac{100}{3}$$

As an example, let’s assume that the original SD for the composite IFABI-R score is 0.5. To standardise this SD on a 100-point scale, we use the formula:

$$\text{Standardised SD}=\text{0.5}\times\frac{100}{3}=16.67$$

Thus, the standardised SD on a 100-point scale would be 16.67.

Figure S3. **Standardised Overall Caregiver QoL Score, by Asthma Severity and Control Levels (PACQLQ questionnaires only)**

Figure S4. **Standardised Overall Caregiver QoL Score, by Asthma Severity and Control Levels (WHO-BREF questionnaires only)**
